# Quantitative simulation and verification of the tourism economic resilience in urban agglomerations

**DOI:** 10.1038/s41598-023-46166-0

**Published:** 2023-11-01

**Authors:** Shasha Yang, Yugui Lu, Shuyue Wang

**Affiliations:** 1 School of Business, Guilin Tourism University, Guilin, 541006 China; 2 School of Economy and Trade, Guangxi Vocational Normal University, Nanning, 530007 Guangxi China

**Keywords:** Socioeconomic scenarios, Sustainability

## Abstract

The concept of tourism economic resilience emphasizes the sustainable development level of tourism economy under uncertainty and risk. Focusing on urban agglomerations, this study aims to describe how the tourism economic resilience is developing, explore whether the resilience level is enhanced with urban agglomerations and whether spatial elements affect resilience levels. With the combination of the aggregation and diffusion effects and crowding effects of regional development, the study uses a combination of dynamic evaluation method, spatial kernel density, and mathematical models of urban agglomeration development to quantitatively analyze the spatiotemporal dynamic evolution of tourism economic resilience from 2006 to 2019, simulates and verifies its development patterns. The conclusions show that: (1) The tourism economic resilience in urban agglomerations is closely related to regional development and prosperity; (2) The development of tourism economic resilience also follows the spatial economic development pattern which moves towards equilibrium in aggregation process; (3) The tourism economic resilience of urban agglomerations has a fluctuation climbing node, generally presents as a wave-like upward trend with fluctuations and stages; (4) The evolutionary trend of tourism economic resilience in urban agglomerations presents as a slight wave-like upward curve that changes with time and co-opetition.

## Introduction

In China, sustainable development is gradually replacing high-speed growth in the economic system. As one of the strategic pillar industries in China, achieving sustainable development transformation in the tourism economy is an inevitable choice for the tourism industry to respond to the national policy call and enhance its overall competitiveness^1^. The structure and function of China's tourism industry have always been affected by uncertainty risks during its development process, thereby threatening tourism economy’s sustainability development^2^. Due to its environmental sensitivity, the tourism economy is vulnerable to fluctuations in natural, social, political, and economic changes within the region, reflecting the fragility of the tourism economy under external impacts^3^. As a vital embodiment of the self-regulating and recovery ability of the comprehensive system, the level of resilience can not only highlight the anti-fragility level of the industry at the implicit level but also explicitly measure the sustainable development level^4^. Cross-discussed the concept of resilience and the tourism economy’s sustainable development, it is a valuable entry point for the tourism economy’s recovery in the post-epidemic era at the macro level and the coordinated development between regions at the meso level. With the advancement of internet technology and transportation accessibility, the economic and cultural exchanges among regions in China are getting increasingly close, the development of the tourism economy also reflects vital regional openness and industrial relevance. Under the background of regional spatial connection increase, the compatibility and integration degree between the tourism economy and regional development are also gradually rising in China^5^. Based on the guiding principle of the sustainable tourism economy, except the remote tourist attraction from outside of the region, potential tourism consumption demands within the area should also be valued and explored^6^. When uncertainty risks such as liquidity constraints emerge, the spatial transformation and substitution between remote and local tourists can provide resilient survival space for the tourism economy^7^. Meanwhile, urban agglomeration is not only a crucial spatial carrier for the new urbanization process but also a concentrated area for the tourism economy development and fierce market competition under the current regional development system in China^8^. Therefore, viewing from the point of urban agglomerations, quantitatively analysis the actual situation of tourism economic resilience, discusses the synchronous evolution process and spatiotemporal influencing factors of tourism economic resilience, which have a significant practical value for the tourism industry to transform from scale and speed-oriented growth to quality and efficiency-oriented development in the new era, then ultimately achieving the maximization of social surplus.

Various types of uncertainty risk obstacles always accompany the economic development of human society. Using resilience criteria to represent the sustainable development ability of economic subjects is currently the mainstream and prevailing evaluation method for most of the government and academics^9^. Through the development process of resilience research, resilience theory was first introduced into applied economics in 2002, which believed that differences in resilience are the key explanatory factors for different outcomes under the same impact in different regions^10^. Since then, the definition of economic resilience has gradually become more apparent. Scholars generally agree that it refers to the ability of an economic system to adapt and restructure its internal structure to mitigate the impact of shocks, while maintaining continued development and utilizing external shocks to achieve system renewal^11^. Thus, the understanding of the concept has gradually shifted from resilience thinking under the framework of the ecological environment to evolutionary resilience thinking, which represents dynamic adaptation and sustainable development goals^12^. However, as an extension of economic resilience, the study of tourism economic resilience has appeared relatively late. Cross-disciplinary exploration of tourism economic resilience mainly focuses on crises’ external impact with natural or social attributes on the tourism economic system and the internal conditions that foster resistance against external impact^13^. Research topics are divided into three different frameworks: ecological environment^14^, socio-economic^15^ or political-geographical conflicts^16^, each related to different types of external impacts. Under the context of ecological and environmental changes, research on the resilience of tourism economies often considers the ecological environment as the foundation for the development of tourism, while climate change, ecological destruction, and environmental pollution can seriously threaten the sustainable development capacity of tourist destinations and expose their vulnerability^17^. As a result of excessive pursuit of short-term benefits, ecological pressure has risen dramatically, disrupting the diversity and balance of the ecological structure, which is the primary source of vulnerability for tourist destinations^18^. Additionally, under the context of socio-economic issues, research at the economic level mainly focused on how large-scale economic crises affect the volatility of tourism economies, and policy recommendations often emphasize the diversity and breadth of tourism products and the broadness of tourism consumption^19^. At the social level, sudden public health events can cause personnel mobility and have become the most discussed topic among numerous socio-economic issues^20^. In particular, the tourism economies stagnated after the COVID-19 pandemic, the anti-fragile properties and evaluation of the regional tourism economies resilience have gradually gained attention^21^. Finally, under the context of political and geopolitical conflicts, resilience research is relatively rare, and the limited research topics are mainly concerned with the impact of war conflicts or terrorism on personnel mobility, security stability, and economic activity in tourist destinations^2^. Such literature often takes terrorist attacks or short-term geopolitical conflicts as an entry point and proposes that conflict risks can affect tourism decision-making at the micro level but have little long-term impact on cross-regional tourism at the macro level. Besides the research topics, tourism economic resilience research methods primarily rely on qualitative studies, with relatively fewer quantitative studies. Among qualitative methods, the research use case analysis, interviews, and questionnaires. For instance, Ntounis et al. (2022) analyzed the factors affecting resilience under the impact of the pandemic using the PRISMA method. Similarly^22^, Quang et al. (2022) used a questionnaire survey to summarize the perceptions of the pandemic’s impact on Vietnam’s tourism industry and provided practical recommendations to enhance the tourism economy resilience further^23^. In terms of quantitative research, methods are more commonly used for evaluating resilience and comparing driving factors. For example, by calculating conditional value at risk, Shi et al. (2023) revealed the importance of providing tourism products, services, and infrastructure in the tourism economic resilience evaluation system^24^. Similarly, Zhang et al.^25^ explored whether the Chinese tourism economy exhibits resilience under different risk cycle contexts and ultimately identified the factors affecting tourism economic resilience through geographic detectors.

The previous studies expanded the research perspectives on tourism economic resilience from multiple angles, providing a matter though for further innovative extensions in this paper. By affirming and using some of the above research perspectives and methods, this article also recognizes the gaps existing in the current literature. Firstly, although some scholars recently have begun the quantitative analysis of tourism resilience, the application of resilience assessment in the tourism economy is still in its infancy, and the guiding theory of the indicators selection still mainly focuses on the restorative and equilibrium aspects within the ecological resilience concept^26,27^, did not take the economic attributes of the tourism industry into the resilience framework. Whether the existing evaluation system can accurately characterize the resilience level of the tourism economy, the application scope, and the inherent value remains to be further verified. Secondly, the current tourism economic data in China are mainly at the national and provincial levels, which is challenging to obtain tourism-related data at the city level from the statistical yearbooks. As a result, quantitative analysis of the tourism economy at the mesoscopic level is relatively few, and a complete study on spatiotemporal evolution patterns of inter-regional tourism economic resilience is lacking. As an essential spatial carrier of factor agglomeration in China at the meso scale, the stable growth of the tourism economy has great practical significance for regional employment issues and sustainable development goals. However, existing research has taken chiefly a macro or micro perspective^28,29^, with few articles choosing urban agglomerations as the entry point for analyzing tourism economic resilience, resulting in a need for more hierarchy and comprehensiveness in the existing field of tourism economic resilience research. Furthermore, scholars have yet to analyze the spatiotemporal evolution pattern of tourism economic resilience from a dynamic perspective, nor have they cross-discussed urban agglomerations development with the development law of tourism economic resilience. With the progress of regional exchanges and the gradual breaking of boundary effects, urban agglomerations have become increasingly important in Chinese regional development. But in terms of regional development, whether the resilience of the tourism economy will rise synchronously with the pace of urban agglomeration development, this question has not received sufficient attention in the field of tourism.

Therefore, by taking the urban agglomerations as the research area, this paper quantitatively evaluates the tourism economic resilience based on a multi-source data structure, characterizes its spatiotemporal pattern features from a dynamic perspective, and finally combines the formation and development process of urban agglomerations with tourism economic resilience development for cross-discussion, to enrich the diversity of tourism economic resilience research in theory and help urban agglomerations plan for sustainable tourism economic development in practice.

## Theoretical frameworks

Tourism economic resilience means the ability of the tourism ecosystem to absorb or withstand disturbances and other pressures, enabling the tourism industry development to maintain its structure and function^30^. Effective tourism resilience management can restore the tourism products supply and the functionality of tourism services at the fastest rate possible through reasonable utilization of internal and external resources^31^. Using dynamic agglomeration economic theory and evolutionary economic geography theory, this article will examine a detailed analysis of the rising pattern of tourism economic resilience in urban agglomerations. Firstly, the dynamic agglomeration economic theory explains the relationship between the tourism economy and spatial expansion of cities within a regional development process from the perspectives of agglomeration and growth. The economic benefits produced by the spatial agglomeration of factors continuously attract the collaboration of surrounding cities with the central city, thereby promoting urban agglomeration’s formation and development, and the tourism economy of urban agglomeration also continuously rises under the agglomeration effect. However, when the scale of tourism economy agglomeration expands to a certain extent along with the region spatial expansion, a congestion effect will appear^32^. Under the combined effects of agglomeration and congestion, tourism economic resilience development will experience some fluctuations. Secondly, from a regional coordinated development perspective, integrating new cities into the development phase of the central city usually leads to a transitional stage characterized by antagonistic reactions, which is reflected as a wave-like upward trend with fluctuations and periodicity^33^. Therefore, under the background of regional co-opetition, there objectively exists a threshold value to determine whether the central city can integrate resources with new cities and thus enhance the tourism economy resilience. Meanwhile, the development stage and tourism economic level in urban agglomerations are quite different in China, which may lead to the deviation of the resilience development trend, and there is a certain spatial and temporal difference. In this regard, this paper will also conduct an in-depth discussion on the spatiotemporal pattern characteristics of the tourism economic resilience from the dynamic perspective.

This article draws on the climbing mechanism and initial formula for sustainable development of urban clusters set up by Fang et al.^34^, and propose the climbing law of the tourism economic resilience of urban agglomeration, then create a wave-like climbing curve of the tourism economic resilience. Different from the previous studies, the climbing law presented in this article intends to the developmental characteristics of tourism economic resilience. Which means, as urban agglomeration develops, there is constant co-opetition among internal tourism economies. However, during the antagonistic stage, the tourism economy is difficult to improve substantially or even experiences a fall in resilience growth. The overall development pattern presents a wave-like climbing form, similar to a series of climbing waves. This is the climbing law followed by tourism economic resilience through urban agglomerations’ development and formation. Exploring this development law, it can provide better service for the layout planning of the sustainable tourism economic development in urban agglomerations, which has important reference value for the tourism industry transformation in from scale and speed-oriented development to quality and benefit-oriented development and from extension and expansion-oriented development to core-connotation-oriented development in the new era.

By further considering the resilience level of core cities during the initial development, this paper has improved the climbing function curve simulation formula. Specifically, it can be expressed as follows:

1 $${P}_{t}={P}_{0}+k\left(t-{t}_{0}\right)+\{{e}^{\left|\alpha \mathrm{sin}\left[\beta \left(t-{t}_{0}\right)\right]\right|}-1\}$$

Variable ${P}_{t}$ indicates the initial resilience potential of the urban tourism economy at time ${t}_{0}$, where ${t}_{0}$ is the initial time, and ${P}_{0}$ represents the initial value. $k$ indicates the slope of the linear function, $\alpha$ represents the amplitude of the trigonometric function, and $\beta$ indicates the periodicity coefficient. In the climbing curve, the climbing rate represents the improved speed of the tourism economic resilience co-opetition in urban agglomeration. By derivation of the above curve formula, the climbing rate can be obtained, which can be expressed as:

2 $$  p_{t}^{\prime }  = \left\{ {\begin{array}{*{20}l}    {k + \alpha \beta \cos \left[ {\beta \left( {t - t_{0} } \right)} \right]e^{{\alpha \sin \left[ {\beta \left( {t - t_{0} } \right)} \right]}} ,t \in \left[ {\frac{{2k\pi  + \beta t_{0} }}{\beta },\frac{{\pi  + 2k\pi  + \beta t_{0} }}{\beta }} \right]} \hfill  \\    {k - \alpha \beta \cos \left[ {\beta \left( {t - t_{0} } \right)} \right]e^{{ - \alpha \sin \left[ {\beta \left( {t - t_{0} } \right)} \right]}} ,t \in \left[ {\frac{{\pi  + 2k\pi  + \beta t_{0} }}{\beta },\frac{{2\pi  + 2k\pi  + \beta t_{0} }}{\beta }} \right]} \hfill  \\   \end{array} } \right.  $$

In the formula, $p_{t}^{\prime }$ is the climbing rate, representing the improvement speed of co-opetition in the tourism economic resilience in urban agglomeration.

## Research design

### Research area

**Figure 1:**
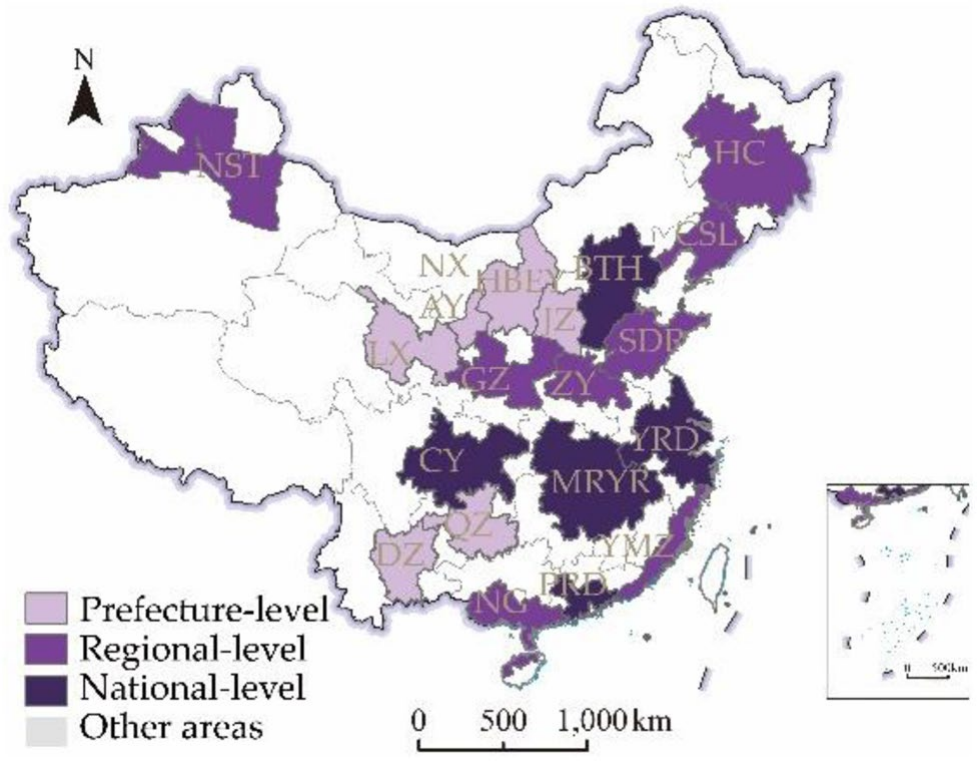
Distribution of urban agglomerations in China. Source: Standard map service website of the Ministry of Natural Resources of China (http://bzdt.ch.mnr.gov.cn/)1:48000000 standard map, approved no. GS(2019)1825. The base map without modification.

Urban agglomerations have gradually become an essential spatial carrier for tourism development in China^35^. Accurately understanding the spatiotemporal pattern characteristics and development rules of tourism economic resilience, it is valuable for promoting the sustainable development of the tourism economy and coordinated development between regions. Therefore, this paper selects 19 urban agglomerations mentioned in China's latest development plans, including National-level urban agglomerations (Yangtze River Delta, Pearl River Delta, Beijing-Tianjin-Hebei, Middle Reaches of Yangtze River, Chengdu-Chongqing); Regional-level urban agglomerations(Shandong Peninsula, Guangdong-Fujian-Zhejiang, Central Plains, Guanzhong Plain, Central- southern Liaoning, Harbin-Changchun, Northern Gulf, Northern Slope of Tianshan Mountains); Prefecture-level urban agglomerations (Dian Zhong, Qian Zhong, Jin Zhong , Lanzhou-Xining, Huhhot-Baotou-Yulin, Ningxia along the Yellow River), with a total of 203 cities as the research object, shown in Fig. 1.

### Research methods

#### Combinatorial dynamic analysis

The traditional methods of Combinatorial evaluation typically rely on static foundations to proceed. In contrast, combinatorial dynamic evaluation introduces time factors from an emotional perspective into the combinatorial evaluation method to ensure continuity of the evaluation results^36^. In order to break through the limitations in existing research, this paper uses the vertical-horizontal stratification method to weigh the fundamental indicators, then uses the fixed base efficiency coefficient method to standardize the indicators and calculate the tourism economic resilience index at the end.

1. Vertical-horizontal stratification method. The vertical-horizontal stratification method is a novel dynamic comprehensive evaluation approach characterized by its straightforward principles, clear intuitive meaning, and transparent evaluation process. It aims to determine weight coefficients based on the differences observed among evaluated objects in a temporal and spatial data table^37^.
  3 $$ \begin{gathered} {\text{ma}}xw^{T} {\text{H}}w \hfill \\ s.t.w = 1 \hfill \\ w > 0 \hfill \\ \end{gathered} $$
  In the formula, it is assumed that the following set of evaluated objects is $s = \left\{ {s_{1} ,s_{2} , \cdots ,s_{n} } \right\}$, and the weight coefficient vector of the indicators is $w = (w_{1} ,w_{2} , \cdots ,w_{m} )^{T}$. $x_{ij} \left( {t_{k} } \right)$ represents the original data of indicator $j$ of the urban agglomeration $i$ in year $t_{k}$. For the moment $t_{k}$, the comprehensive evaluation function is defined as $y_{i} \left( {t_{k} } \right) = \mathop \sum \nolimits_{j = 1}^{m} w_{j} x_{ij}^{*} \left( {t_{k} } \right)$, and the total sum of squares of deviations between $y_{i} \left( {t_{k} } \right)$ and the mean value $\overline{y}$ is $\sigma^{2} = \mathop \sum \nolimits_{k = 1}^{N} \mathop \sum \nolimits_{i = 1}^{n} \left[ {y_{i} \left( {t_{k} } \right) - \overline{y}} \right]^{2}$, which measures the differences among the evaluated objects. Normalizing the original data can be assumed as $\sigma^{2} = \mathop \sum \nolimits_{k = 1}^{N} \mathop \sum \nolimits_{i = 1}^{n} \left[ {y_{i} \left( {t_{k} } \right)} \right]^{2} = \mathop \sum \nolimits_{k = 1}^{N} \left[ {w^{T} H_{k} w} \right] = w^{T} \mathop \sum \nolimits_{k = 1}^{N} H_{k} w = w^{T} Hw$. Here, $H = \mathop \sum \nolimits_{k = 1}^{N} H_{k}$ is an $\times m$ $H_{k} = A_{k}^{T} A_{k}$. Assuming $w^{T} w = 1$, $w$ can be chosen as the eigenvector corresponding to the largest eigenvalue of matrix $H$, and $\sigma^{2}$ takes the max value. To ensure that all weight coefficients are positive, it can be further assumed that $w > 0$, and the weight coefficient vector of the indicators $w$ can be obtained through the optimization problem in the above formula.
2. Fixed base efficiency coefficient method. In order to effectively compare tourism resilience across different years, this study employs the fixed base efficiency coefficient method to standardize the original data using 2006 as the base year. It is a normalization technique that utilizes efficiency coefficients to account for changes in the level of input and output variables over time^38^. The specific formula used in this study is notated as follows:
  4 $$ s_{ij} \left( {t_{k} } \right) = 10 \times \frac{{\max \left[ {x_{j} \left( {t_{1} } \right)} \right] - x_{ij} \left( {t_{k} } \right)}}{{\max \left[ {x_{j} \left( {t_{1} } \right)} \right] - \min \left[ {x_{j} \left( {t_{1} } \right)} \right]}} $$
  In the formula, indicator $j$ in urban agglomeration $i$ is represented by the raw value and standard value in year ${t}_{k}$, denoted as ${x}_{ij}\left({t}_{k}\right)$ and ${s}_{ij}\left({t}_{k}\right)$, respectively.
3. Linear weighting method: Combining the weight coefficient vector mentioned above with the standardized indicator values, the paper uses it to calculate the tourism economic resilience index, denoted as ${Q}_{i}\left({t}_{k}\right)$, for $i$ urban cluster in the year ${t}_{k}$. The formula is presented as follows:
  5 $$ Q_{i} \left( {t_{k} } \right) = \mathop \sum \limits_{j = 1}^{m} w_{j} s_{ij} \left( {t_{k} } \right) $$

#### Spatial kernel density analysis

Compared to traditional kernel density estimation, spatial kernel density has a broader perspective and can better reflect the spatial dynamic characteristics of the analysis object^39^. Based on the ordinary kernel density, the paper estimates the probability density of random variables by adding time and space parameters. In the formula, $f\left(x,y\right)$ is the joint kernel density function of $x$ and $y$, $g\left(y|x\right)$) is the distribution of $y$ under the condition of $x$.

6 $$ f\left( {x,y} \right) = \frac{1}{{nh_{x} h_{y} }}\mathop \sum \limits_{i = 1}^{n} K_{x} \left( {\frac{{X_{i} - x}}{{h_{x} }}} \right)K_{y} \left( {\frac{{Y_{i - } y}}{{h_{y} }}} \right) $$

7 $$ g\left( {y|x} \right) = \frac{{f\left( {x,y} \right)}}{f\left( x \right)} $$

#### Co-opetition and threshold value calculation

Firstly, co-opetition intensity means the degree to which tourism economies of cities attract and integrate with each other. It is positively correlated with resilience level and negatively correlated with spatial distance^40^. With the development of urban agglomeration, the intensity can appropriately reflect the competitive process of the tourism economic resilience^41^. According to the climbing law and the basic function model, we have developed a formula for calculating the intensity of co-opetition in urban agglomeration. The specific formula is as follows:

8 $${F}_{xy}\text{=}\frac{\sqrt{{C}_{x}\times {C}_{y}}\times \sqrt{{\text{G}}_{x}\times {\text{G}}_{y}}}{{\text{D}}_{\text{xy}}^{2}}\times \frac{1}{100}$$

${F}_{xy}$ denotes the co-opetition intensity of resilience between cities $x$ and $y$, while ${C}_{x}$ and ${C}_{y}$ denote the resilience level respectively. ${\text{G}}_{x}$ and ${\text{G}}_{y}$ represent the GDP of cities $x$ and $y$, respectively, ${\text{D}}_{\text{xy}}^{2}$ denotes the distance between cities $x$ and $y$.

Furthermore, during the process of tourism economic resilience enhancement in urban agglomerations, there is a transition phase in which mutual attraction and co-opetition between cities coexist in an antagonistic state^42^. The overall trend exhibits a wave-like fluctuation and periodicity, and there is an objective threshold value of co-opetition to determine whether the curve of the tourism economy resilience has entered the next stage due to co-opetition-induced changes in the resilience level of cities.

Drawing on Duan & Tang, (2022)'s method for setting threshold values in the analysis of the coordinated development process within urban agglomerations, this paper uses the mean of the co-opetition intensity as a threshold value for determining whether to unite with a new city. The formula is presented as follows:

9 $$ \lambda_{xy} = \frac{1}{2} \times \frac{{\mathop \sum \nolimits_{x = 1}^{m} \mathop \sum \nolimits_{y = 1}^{n} F_{xy} }}{x \times y} $$

In the formula, ${\uplambda }_{{{\text{xy}}}}$ denotes the threshold value of co-opetition between cities; ${\text{F}}_{xy}$ stands for the intensity, ${\text{x}}$ is the number of years, and ${\text{y}}$ is the number of cities.

### Index system

Different from the current resilience conceptual framework, which usually based on ecological thinking, this article constructs a resilience evaluation system starting from the economic attributes of the tourism industry and proposes that tourism resilience should focus on the degree of integration and compatibility between the tourism industry and the regional economy. The sustainable development of the tourism economy not only relies on tourists from outside the region but also needs to tap into the potential demand of local residents^43^. When uncertainties such as mobility restrictions arise, the transformation and substitution of remote and local tourists can provide resilience survival space for the tourism economy^44^. Tourism activities is the core of the tourism economy. The tourism economy is vulnerable to risks due primarily to the strong tourism demand of tourists and the high supply capacity among tourism service providers^45^. Furthermore, enriching the structure of the tourism economy and strengthening the government's management capacity is essential to enhance the resilience of tourism activities in response to social risk shocks^46^. Accordingly, this article will start from the economic attributes of the tourism industry and evaluate its resilience from four perspectives: supply, demand, structure, and management.

In the framework, supply resilience and demand resilience comprehensively consider the business anti-vulnerability of tourism destinations under the liquidity crisis, which can also refer to the integration degree between the tourism industry and the local economy^21^.

In accordance with Calgaro et al.^47^ and Prayag et al.^48^, this paper characterizes the tourism economy supply resilience with indicators such as the number of star hotels, number of tourist attraction, etc., which measures the ability of tourism service providers to recover to the desired state after being disturbed by risks. Taking Sharma et al.^49^’s study into account, this paper characterizes the tourism economy demand resilience with indicators like the scale of tourist number, tourism consumption level, etc., which measures the motivation of tourists to resume tourism activities after being disturbed by risks. Structural resilience mainly considers whether the tourism destination has the adjustment and innovation ability to form digital tourism or alternative tourism under uncertain risks such as liquidity restriction or consumption trend change^20^. According to the studies of Lu et al.^50^and Anguera-Torrell et al.^51^, this paper characterizes the tourism economy structural resilience with indicators such as the industrial structure rationalization index, the proportion of employees in the digital industry, etc., which measures whether tourism destinations can adjust to the formation of digital tourism instead of traditional tourism after being disturbed by risks. Management resilience is represented by the investment status and importance of tourism destinations in tourism ecological environment protection, tourism industry support, infrastructure support, and other aspects, which reflects the potential support ability of tourism destinations for the anti-vulnerability of the tourism economy^52^. Taking into account the findings of Wang et al.^53^, this paper characterizes the tourism economy managerial resilience with indicators like the intensity of environmental regulation, tourism industry attention level, etc., which measures the potential coping capacity of tourism destinations after being disturbed by risks. The specific framework and indicators are shown in Table 1.

**Table1:** Evaluation index system of tourism resilience.

A	B	C	D
Target layer	Criterion layer	Indicator layer	Calculation method
Resilience of tourism economy	Supply resilience	Contribution rate of tourism	Regional tourism economic revenue /GDP
		Number of star hotels	–
		Number of tourist attraction	–
		The proportion of workers in accommodation and catering industry	Number of employed people in accommodation and catering industry/total number of employed people in the region
		The proportion of employees in the cultural and entertainment industries	Number of employed people in cultural and entertainment industries/total number of employed people in the region
		Night light intensity	–
	Demand resilience	Scale of tourist number	–
		Tourism consumption level	Regional tourism economic income/regional tourist number
		Tourism income	–
		Population density	–
		Disposable income per capita	Total disposable income/total population of the region
		Highway passenger volume	–
	Structure resilience	Industrial structure rationalization index	Regional tertiary industry output value/secondary industry output value
		Efficiency of capital allocation	Loan balance of regional financial institutions/deposit balance of financial institutions
		The proportion of employees in digital industry	Number of employees in digital industry/total number of employed people in the region
		Proportion of green innovation patents	Number of regional green innovation patents/total number of innovation patents
		Degree of market openness	
	Management resilience	Total investment in fixed assets	–
		Intensity of environmental regulation	District governments report environment-related word frequency/total word frequency
		Government intervention level	Regional fiscal expenditure /GDP
		Social security, employment expenditure	–
		Tourism industry attention level	Regional governments report tourism related word frequency/report total word frequency

### Data range and sources

Since China proposed the concept of urban agglomeration construction begun from 2006, and most of the current data related to the tourism economy has been updated until 2019, this paper now selects the study period from 2006 to 2019 as the research timeframe.

The spatial vector data. In this study, basic map vector data are derived from the national basic geographic information database, and uses ArcGIS software to determine the distribution of urban agglomeration.

Platform sharing data. The green innovation patents, population density, number of star-rated hotels, tourism volume, and economic income data used in this paper were obtained from the Chinese Research Data Services (www.cnrds.com) and the Patent Retrieval and Analysis System of the National Intellectual Property Administration. This paper mainly uses indicators such as tourism volume, tourism economic income, and population density to reflect the resilience of remote and local tourism economic demand.

Portal data. This paper collects tourism industry attention and environmental regulation intensity data from local government portals. The ratio of tourism word frequency to total report word frequency and environmental word frequency to total report word frequency respectively reflects the level of tourism industry attention and environmental regulation intensity, which is used as an evaluation index of the resilience level of tourism economic management of tourist destinations.

Scientific sharing data. Since there are no officially published intensity data, this paper obtained the intensity data of nighttime lighting from articles published in the data-sharing journal Scientific Data^54^. In combination with the obtained data on the number of star hotels and other relevant information, this paper aims to comprehensively characterize the scale and diversity of tourism resources and product supply in urban agglomeration.

Regional statistical data. The remaining social-economic statistical data in this paper were obtained from the China City Statistical Yearbook, China Regional Economic Yearbook, and China Construction Yearbook from 2007 to 2020, with a few of missing values supplemented by interpolation averaging.

## Results

### Quantitative analysis of tourism economic resilience in urban agglomerations

**Figure 2:**
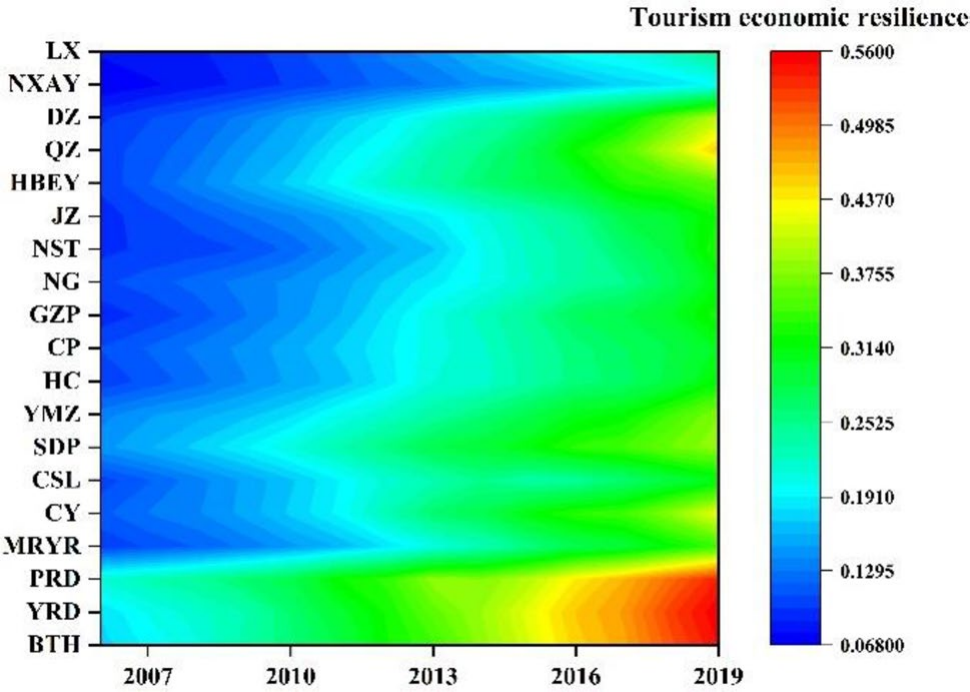
Evolution trends of tourism economic resilience in urban agglomerations.

Tourism economic resilience were quantitatively evaluated by the combinatorial dynamic evaluation method. Now, this paper uses the Origin software to conduct matrix processing on the resilience values. Finally, a Planar heat map is used for visualization, shown in Fig. 2.

From the perspective of resilience distribution, the hierarchical distribution characteristics of the national-level urban agglomerations are more prominent. The resilience index of BTH, YRD, and PRD urban agglomerations exceed 0.5, with an outstanding resilience level performance compared to other urban agglomerations. Moreover,, the resilience index of the YRD urban agglomeration reached 0.56 at the end of the research year, which is the highest. In the regional-level urban agglomerations, the SDP and YMZ urban agglomerations have quickly reached a resilience index of 0.3 and have a trend of crossing into the 0.4 interval, relatively leading in the regional-level urban agglomerations, when other urban agglomerations are primarily around a resilience level of 0.3, and the difference between the agglomerations is not significant. Prefecture-level urban agglomerations are more evident in stages, QZ and DZ urban agglomerations climb to the range of 0.4 at the end, which are in the first echelon. The values of JZ, HBEY, NXAY and LX urban agglomerations are around 0.3 and 0.2, respectively. Among them, the end-stage resilience level of NXAY urban agglomeration is only 0.19, which is the lowest.

In general, four of national-level urban agglomerations are leading the country in tourism economies resilience, with all ranking within the top four. It has not only reflected the dominant position of these urban agglomerations in terms of the tourism economies anti-fragility but also highlights the intrinsic link between the tourism economies resilience and the regional development and prosperity.

From the perspective of resilience climbing, different urban agglomerations show varying levels of improvement. Except LX urban agglomeration shows a slight decline at the early stages, all others have shown a fluctuating upwards. The average climb rate reached 9.4%. Among them, the average increase rate of DZ and QZ urban agglomerations reached 12%, which has the best resilience improvement performance among all urban agglomerations, while PRD urban agglomeration has the lowest increase rate at only 7.4%. Compared to different types of urban agglomerations, prefecture-level urban agglomerations have a lower resilience foundation at the start but have grown rapidly in terms of the increase rate and are gradually narrowing the gap with the regional-level urban agglomerations. Despite this, resilience can still be significantly improved.

In general, high resilience level urban agglomerations have gradually achieved stable average annual growth rates due to the stronger economic foundations and larger scales. However, location factors, lack of tourism resources, or poor regional spatial development benefits primarily restrict the above-mentioned Dianzhong and Qianzhong urban agglomerations with higher growth rates^55^. The fundamental resilience of Dianzhong and Qianzhong urban agglomerations is poor, and their brutal growth is still in its early stages. Upon preliminary observation, the distribution pattern and evolutionary trend of the tourism economic resilience exhibit a strong similarity to Chinese regional economic development laws.

### Spatial–temporal evolution of tourism economic resilience in urban agglomerations

**Figure 3:**
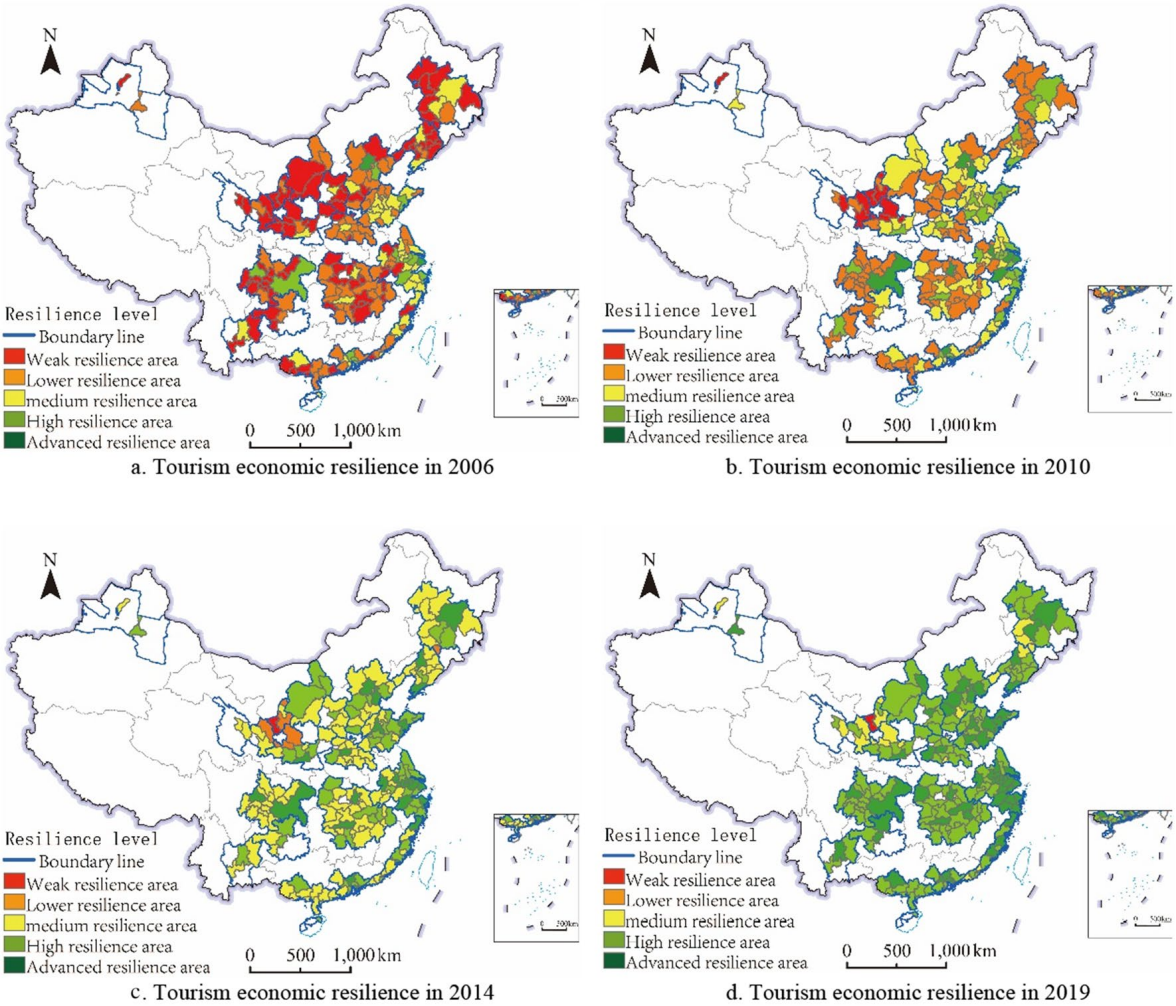
Spatial–temporal evolution of the tourism economic resilience in urban agglomerations. Source: Standard map service website of the Ministry of Natural Resources of China (http://bzdt.ch.mnr.gov.cn/)1:48000000 standard map, approved no. GS(2019)1825. The base map without modification.

To further explore the distribution pattern and evolutionary trend of the tourism economic resilience of each city, this paper employs the natural interval classification method in ArcGIS 10.8 software to classify the resilience level. Meanwhile, this paper also selects four characteristic nodes of 2006, 2010, 2014, and 2019 to facilitate the observation of development changes within the research time frame, shown in Fig. 3.

From the temporal perspective, there is a significant increase in the resilience levels of cities at the national, regional, and prefecture-level urban agglomerations observed during the studied period. The improvement in resilience was particularly pronounced from the early to the late stages of the research period. In 2006, urban agglomerations cities were characterized as weak or lower resilience levels, with only regional central cities exhibiting relatively higher resilience in tourism economies. However, as the study period progressed, there was a notable increase in both the frequency and probability of low-resilience cities transitioning into higher resilience categories. By 2014, the emergence of regionalized distribution of weak resilience level had mainly been resolved, with only a few cities in the NXAY and LX urban agglomeration remaining relatively lower. By 2019, the overall improvement of the tourism economic resilience level in urban agglomerations has achieved initial success. The central cities in the region have basically entered the advanced resilience stage, and the vast majority of surrounding cities have risen to high resilience level, except for a few medium-level resilience cities in the northwest and northeast urban agglomerations.

From the spatial perspective, tourism economic resilience in Chinese urban agglomerations can be classified into three categories. Firstly, the BTH, YRD, and PRD urban agglomerations in the eastern coastal area have the most significant number of advanced resilience cities with relatively strong spatial consistency. Secondly, the resilience levels of the LX and NXAY urban agglomerations at the prefecture level have consistently been low, with less improvement, and have deviated from the overall trend of tourism economic resilience development compared to other urban agglomerations. Except for these, the climbing rate and distribution scale of resilience levels in other urban agglomerations have no significant differences.

In combination with the characteristics of temporal development and spatial patterns, it can be observed that the upward trend of regional tourism economic resilience gradually extends from national-level urban agglomerations in the eastern coastal regions to regional and prefecture-level urban agglomerations in the central and western regions. Within the regions, the development of tourism economic resilience gradually radiates from the central cities towards the surrounding cities. Clearly, there is a correlation between the formation and development of urban agglomeration and increasing regional tourism economic resilience. In urban agglomerations, the spatial pattern and evolutionary law of tourism economic resilience development are basically consistent with the regional economic development, both following the spatial economic development law, which moves from agglomeration to equilibrium.

### Spatial kernel density analysis of tourism economic resilience in urban agglomerations

**Figure 4:**
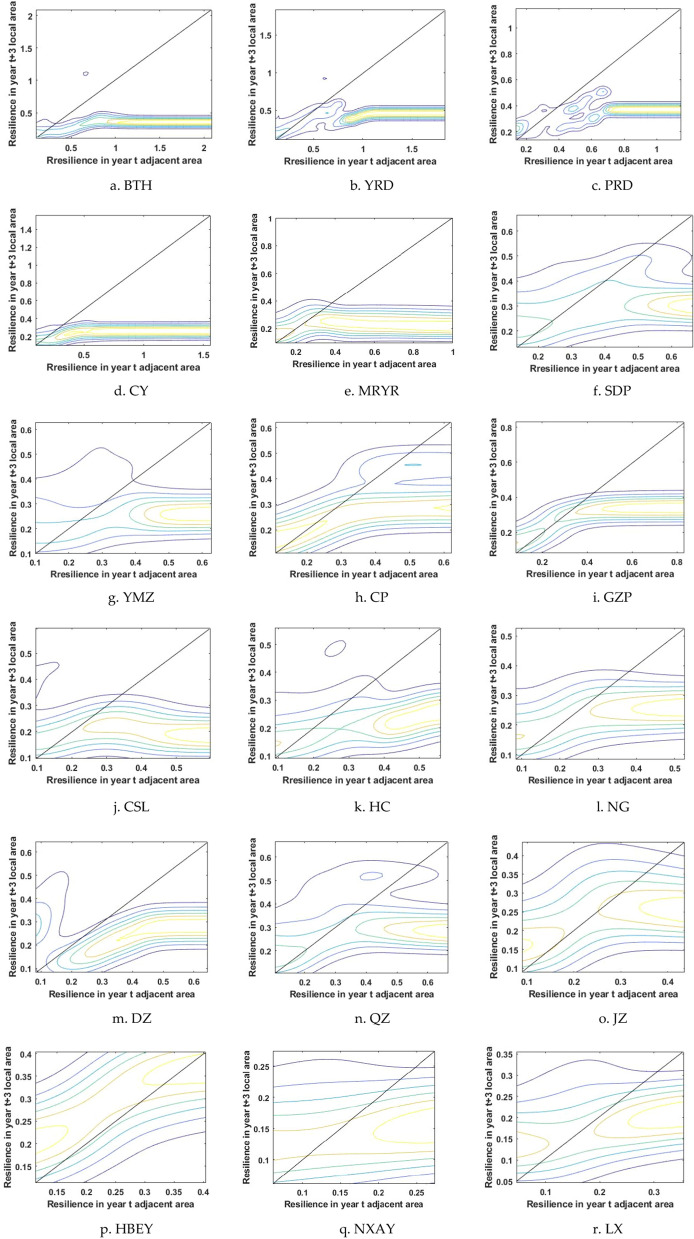
Spatial dynamic kernel density contours of tourism economic resilience in urban agglomerations.

By understanding of the resilience levels and development status in urban agglomerations, this paper further analyzes the spatial distribution pattern and dynamic evolution process of resilience development in urban agglomerations from a dynamic perspective through spatial kernel density model under spatial lag conditions, Fig. 4 shows the impact of the resilience of adjacent regions on the resilience development of the local region in the t + 3 year. The advantage of spatial kernel density lies in the synchronicity of spatiotemporal information, which provides a new perspective for exploring the distribution dynamics and evolution trends of resilience development from a spatiotemporal dimension in this paper. Due to the lack of spatial contiguity between Urumqi and Karamay cities in the NSTM urban agglomeration, this paper excluded it from the spatial matrix construction.

From the perspective of direction, the contour lines of national-level urban agglomerations exhibit a feature of parallelism with the X-axis, indicating strong directional consistency. It has reflected that within the urban agglomeration, the tourism economic resilience in adjacent areas in year t has a more significant impact on the local region in year t + 3. The contour lines of the regional-level and prefecture-level urban agglomerations are generally located near the diagonal line of 45 degrees, indicating that the spatial dynamic spillover effects between adjacent regions and the overall region generally exist in these types of urban agglomeration. Among them, the HBEY urban agglomeration has the contour lines of density closest to the diagonal, showing the most robust positive spatial dynamic spillover effect. Other urban agglomerations tend to turn towards the negative diagonal, suggesting that in such urban agglomerations, when resilience reaches a high level, there is a hidden risk of negative spatial dynamic spillover impact from adjacent areas.

From the perspective of distribution, except MRYR urban agglomeration exhibits a strong main peak and a weak side peak distribution pattern, while all other national and regional level urban agglomerations show a single-peak distribution pattern with a fine integrated shape. It shows that the spatial distribution of tourism economic resilience in most national and regional level urban agglomerations are similar, reflecting a relatively balanced resilience development trend within the agglomeration. In contrast, among the prefecture-level urban agglomerations, except DZ and NXAY urban agglomerations, all others exhibit a bimodal distribution pattern, indicating a differentiated trend in the development of resilience within the agglomerations.

In general, national-level urban agglomerations benefit from advantages in geographic location, economic structure, and the depth and breadth of tourism economies, resulting in the highest level of stability in resilient development. These agglomerations are less susceptible to negative spatial spillover effects from neighboring regions. However, regional-level and prefecture-level urban agglomerations are prone to negative spillover effects and easy to encounter development bottlenecks when the resilience level reaches a certain level, this is the change node where the tourism economic resilience of urban agglomeration fluctuates.

### Co-opetition trends analysis of tourism economic resilience in urban agglomerations

**Figure 5:**
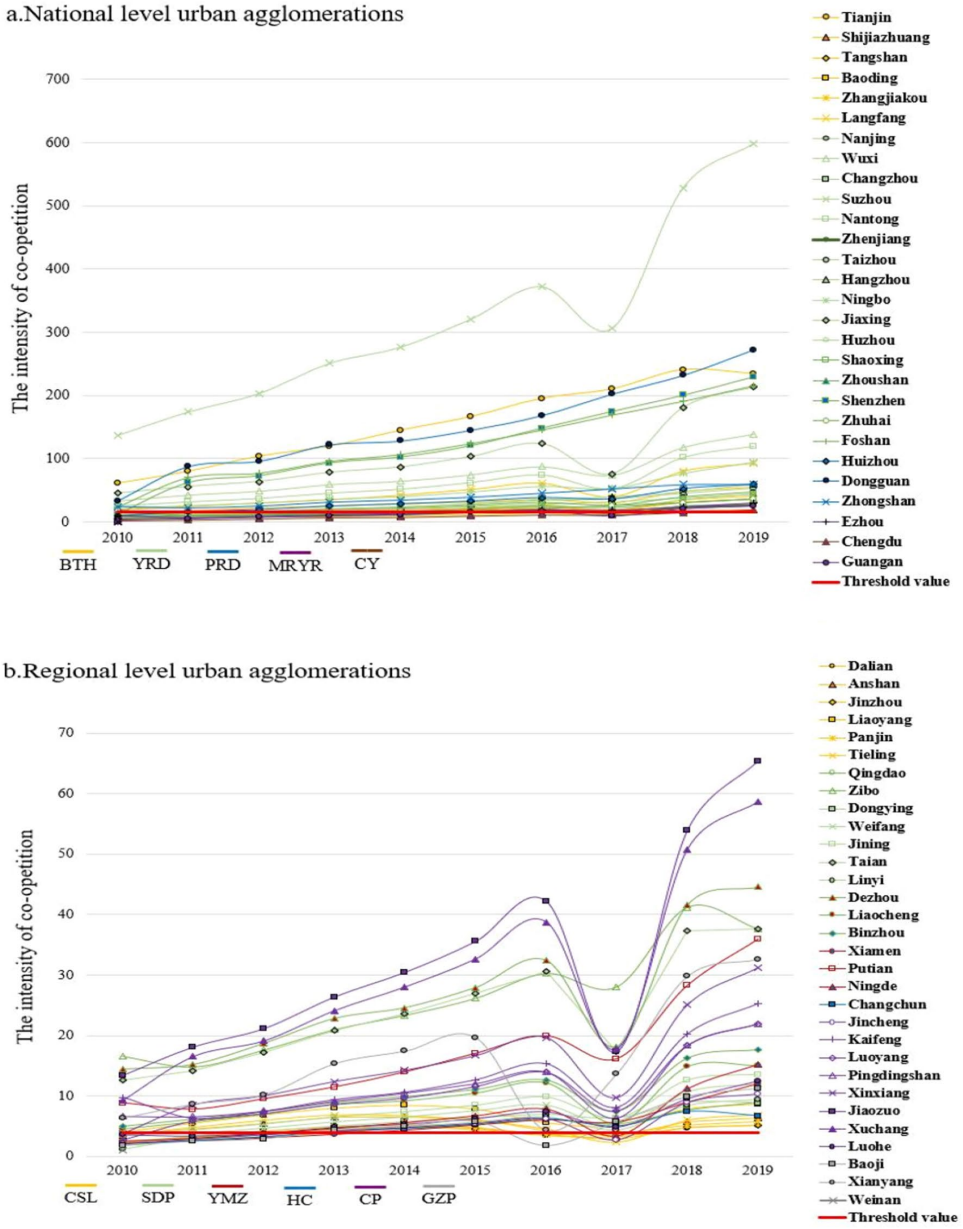

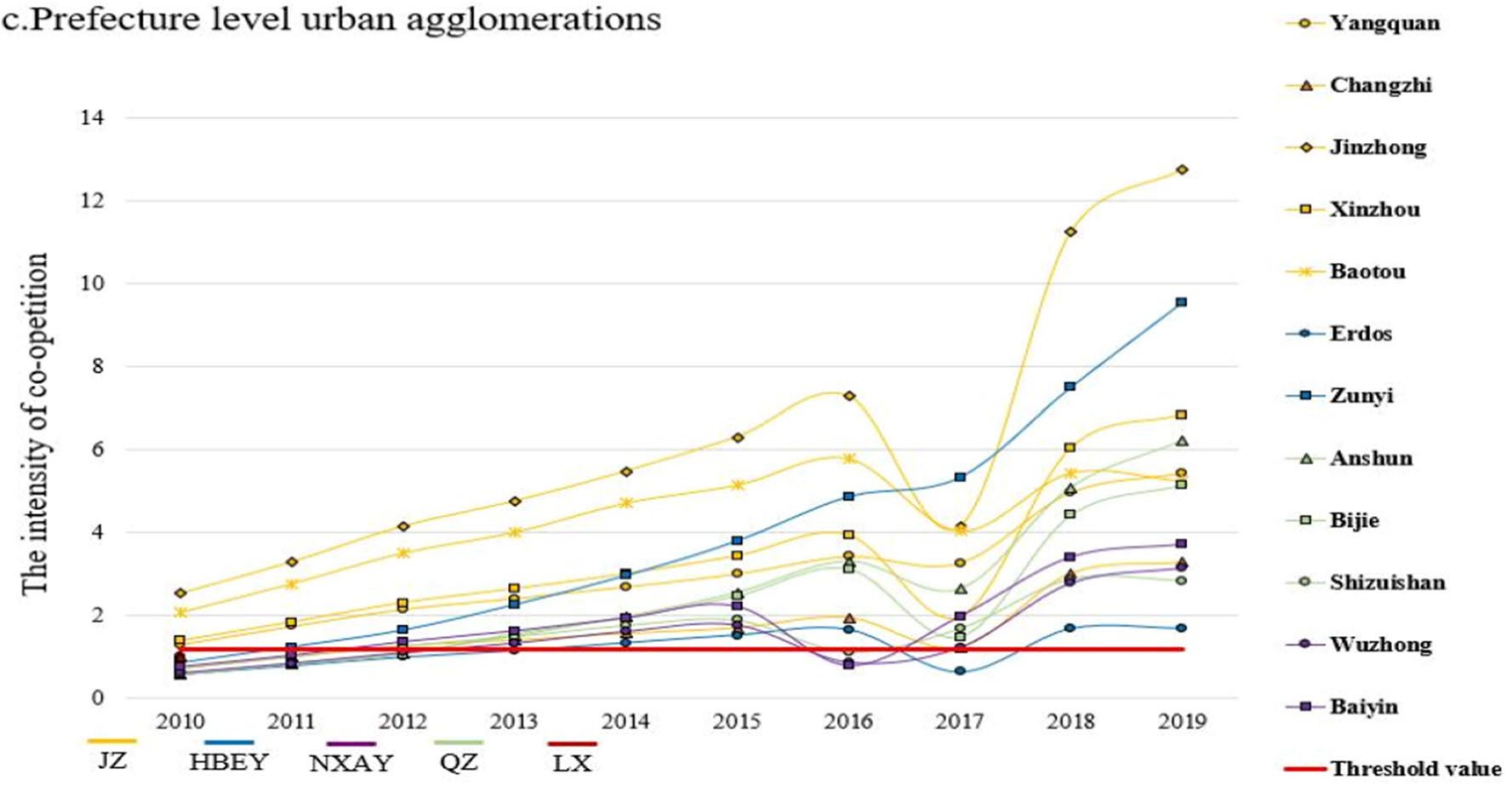
Co-opetition trends in urban agglomerations.

In the context of urban agglomeration formation and development, for a deeper understanding of the competitive and cooperative development trends in tourism economic resilience, formula 8 and formula 9 are used to calculate the co-opetition intensity and the co-opetition threshold value of each type of urban agglomeration. Due to space limitations of the article, this paper only presented the cities with higher than the threshold value, shown in Fig. 5.

From the perspective of intensity comparison, the co-opetition threshold values for national, regional, and prefecture urban agglomeration are 15.34, 3.92, and 1.17, respectively. Among them, there are 28 cities in the national level urban agglomerations whose co-opetition intensity exceeds the threshold value, and the overall intensity, threshold value is both significantly higher than regional and prefecture urban agglomerations. Secondly, there are 31 cities in the regional urban agglomerations whose intensity exceeds the threshold value, which is the most significant number among the three types of urban agglomeration, but the overall level of intensity is relatively low, with fewer high-value cities and more evident internal disparities. Finally, there are only 14 cities in the prefecture-level urban agglomerations that exceeded the threshold value, and the overall intensity and threshold height were at the lowest among the three types of urban agglomeration. Except for the Jinzhong city, the co-opetition intensity of other cities was all lower than 10. Overall, the spatial distribution is highly similar to the tourism economic resilience.

From the perspective of development trends, from the perspective of development trends, each urban agglomeration introduced the “13th Five-Year Plan” tourism development plan one after another in 2017, which led to significant changes in the tourism industry. The plan changed the rating standards for travel agencies and hotels and strengthened the supervision of regional environmental protection. As a result, the competition intensity showed a more obvious deviation from the trend changes, and the adjustment was completed quickly in 2018. In addition to this situation, the intensity among the three types of urban agglomeration all basically showed a slightly fluctuating upward trend. However, it should be noted that, except for the national-level urban agglomerations, some cities in the regional-level and prefecture-level urban agglomerations experienced a situation where the co-opetition intensity reached the threshold value, then fell below the threshold value and subsequently rebounded during the research period, with relatively intense volatility. The results suggest that the overall stability of national-level urban agglomerations is relatively strong, but the co-opetition within the regional and prefecture level urban agglomerations are more prone to exhibit antagonistic transitional phases together with urban agglomerations formation and development, which reflected as a fluctuating and periodic pattern of wave-like growth.

### Simulation and verification of the resilience curves for urban agglomerations

**Figure 6:**
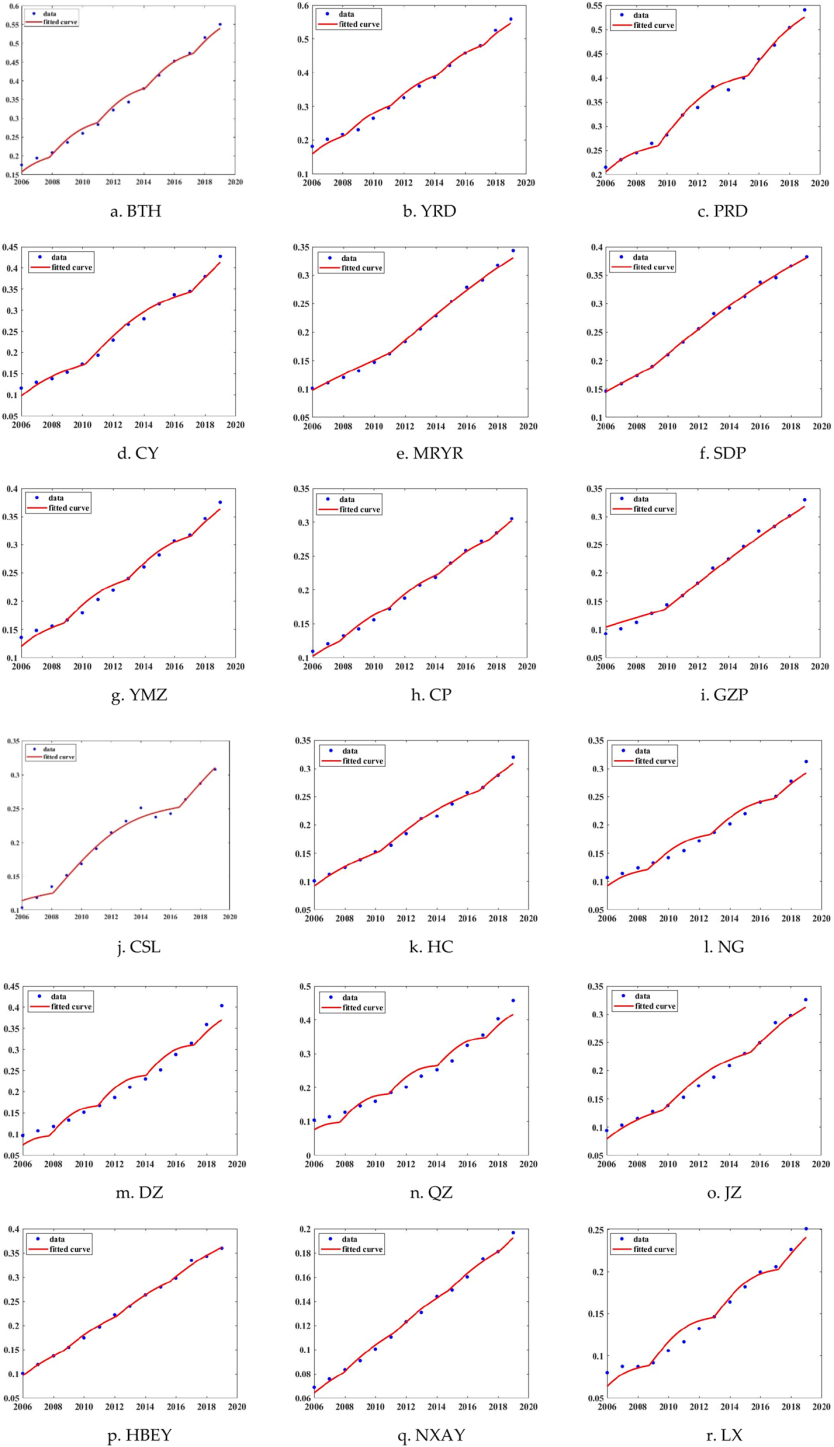
Fitting curve diagram for resilience development law in urban agglomerations.

Finally, based on the quantitative measurement of the tourism economic resilience in urban agglomerations from 2006 to 2019, this paper uses MATLAB software to fit the improved climbing function curve simulation formula. It has passed the chi-square fitting goodness-of-fit test and obtained the optimal functional expression of the tourism economic resilience climbing curve for each urban agglomeration, presented in Table 2. Then, the climbing curve fitting graphs of the tourism economic resilience are drawn. The resulting images provide the most intuitive judgment basis for determining whether the tourism economic resilience conforms to the proposed wave climbing development rule in this paper, which is shown in Fig. 6.

**Table 2:** The optimal function expression of resilience curve for urban agglomerations.

Classification	Urban agglomeration	The optimal function expression
National-level	BTH	${\text{P}}_{{\text{t}}} { = }{ - }{59}{{.99 + 0}}{.02963*(\text{t} + 24}.17) + \{\text{ e}^{{\left| { - 0.001286{\text{*sin}}\left( {0.6659{*}\left( {{\text{t}} + 24.17} \right)} \right)} \right|}} { - }{{1}}\}$
	YRD	${\text{P}}_{{\text{t}}} { = }{ - }{67}{{.35 + 0}}{{.02958*(\text{t} + 276}}{{.7) + \{ e}}^{{\left| {0.001202{\text{*sin}}\left( {0.6624{*}\left( {{\text{t}} + 276.7} \right)} \right)} \right|}} { - }{\text{1}}\}$
	PRD	${\text{P}}_{{\text{t}}} { = }{ - }{61}{\text{.92 + 0}}{{.02469*({\text{t}} + 508}}{.6) + \{ \text{e}}^{{\left| {0.03084{\text{*sin}}\left( {0.5353{*}\left( {{\text{t}} + 508.6} \right)} \right)} \right|}} { - }{\text{1}}\}$
	CY	${\text{P}}_{{\text{t}}} { = }{ - }{57}{\text{.81 + 0}}.02439*(t + 367) + \{ {\text{e}}^{{\left| {{ }0.01903{\text{*sin}}\left( {0.3195{*}\left( {{\text{t}} + 367} \right)} \right)} \right|}} { - }{\text{1}}\}$
	MRYR	${\text{P}}_{{\text{t}}} { = 1}{ - }{35}{\text{.48 + 0}}{\text{.01928*(t}}{ - }{161}.5) + \{ {\text{e}}^{{\left| {{ }0.004067{\text{*sin}}\left( {1.009{*}\left( {{\text{t}} - 161.5} \right)} \right)} \right|}} { - }{\text{1}}\}$
Regional-level	SDP	${\text{P}}_{{\text{t}}} { = }{ - }{42}{\text{.13 + 0}}{\text{.01917*(t + 198}}.8) + \{ {\text{e}}^{{\left| {0.01301{\text{*sin}}\left( {0.3372{*}\left( {{\text{t}} + 198.8} \right)} \right)} \right|}} { - }{\text{1}}\}$
	YMZ	${\text{P}}_{{\text{t}}} { = }{ - }{34}{\text{.52 + 0}}{\text{.01305*(t + 648}}.9) + \{ {\text{e}}^{{\left| {{ }0.05547{\text{sin}}\left[ {0.2145\left( {{\text{t + 231}}{.4}} \right)} \right]} \right|}} { - }{\text{1}}\}$
	ZY	${\text{P}}_{{\text{t}}} { = }{ - }{31}{\text{.87 + 0}}{\text{.01545*(t + 63}}.2) + \{ {\text{e}}^{{\left| { - 0.00103{\text{*sin}}\left( { - 0.3785{*}\left( {{\text{t}} + 63.2} \right)} \right)} \right|}} { - }{\text{1}}\}$
	GZ	${\text{P}}_{{\text{t}}} { = }{ - }{38}{\text{.51 + 0}}{\text{.01883*(t + 42}}.53) + \{ {\text{e}}^{{\left| { - 0.01177{\text{*sin}}\left( {0.9623{*}\left( {{\text{t}} + 42.53} \right)} \right)} \right|}} { - }{\text{1}}\}$
	CSL	${\text{P}}_{{\text{t}}} { = }{ - }{31}{\text{.88 + 0}}{\text{.01491*(t + 138}}.8) + \{ {\text{e}}^{{\left| {0.02965{\text{*sin}}\left( {0.3484{*}\left( {{\text{t}} + 138.8} \right)} \right)} \right|}} { - }{\text{1}}\}$
	HC	${\text{P}}_{{\text{t}}} { = }{ - }{35}{\text{.39 + 0}}{\text{.01671*(t + 116}}.8) + \{ {\text{e}}^{{\left| {0.01101{\text{*sin}}\left( {0.4829{*}\left( {{\text{t}} + 116.8} \right)} \right)} \right|}} { - }{\text{1}}\}$
	NG	${\text{P}}_{{\text{t}}} { = }{ - }{30}{\text{.09 + 0}}{\text{.01509*(t}}{ - }{6}.05) + \{ {\text{e}}^{{\left| {0.008731{\text{*sin}}\left( {0.9139{*}\left( {{\text{t}} - 6.05} \right)} \right)} \right|}} { - }{\text{1}}\}$
Prefecture-level	DZ	${\text{P}}_{{\text{t}}} { = }{ - }{45}{\text{.45 + 0}}{\text{.02267*(t + 0}}.7178) + \{ {\text{e}}^{{\left| {0.02252{\text{*sin}}\left( {0.7771{*}\left( {{\text{t}} + 0.7178} \right)} \right)} \right|}} { - }{\text{1}}\}$
	QZ	${\text{P}}_{{\text{t}}} { = }{ - }{107}{\text{.3 + 0}}{\text{.03459*(t + 1093)}} + \{ {\text{e}}^{{\left| {0.1577{\text{*sin}}\left( {0.1819{*}\left( {{\text{t}} + 1093} \right)} \right)} \right|}} { - }{\text{1}}\}$
	JZ	${\text{P}}_{{\text{t}}} { = }{ - }{33}{\text{.46 + 0}}{\text{.01796*(t}}{ - }{139}.5) + \{ {\text{e}}^{{\left| {0.01601{\text{*sin}}\left( {0.551{*}\left( {{\text{t}} - 139.5} \right)} \right)} \right|}} { - }{\text{1}}\}$
	HBEY	${\text{P}}_{{\text{t}}} { = - 43}{\text{.38 + 0}}{\text{.02069*(t + 95}}.28) + \{ {\text{e}}^{{\left| {0.003227{\text{*sin}}\left( {0.4358{*}\left( {{\text{t}} + 95.28} \right)} \right)} \right|}} {\text{ - 1}}\}$
	NXAY	${\text{P}}_{{\text{t}}} { = }{ - }{21}{\text{.69 + 0}}{\text{.009852*(t + 201)}} + \{ {\text{e}}^{{\left| {0.01055{\text{*sin}}\left( {0.2059{*}\left( {{\text{t}} + 201} \right)} \right)} \right|}} { - }{\text{1}}\}$
	LX	${\text{P}}_{{\text{t}}} { = }{ - }{28}{\text{.72 + 0}}{\text{.01351*(t + 124}}.1) + \{ {\text{e}}^{{\left| { - 0.01383{\text{*sin}}\left( {0.7439{*}\left( {{\text{t}} + 124.1} \right)} \right)} \right|}} { - }{\text{1}}\}$

Upon the observation of Fig. 6, it shows that all the evolutionary curves of the tourism economic resilience have a good fitting effect. In terms of the overall trend, it is in line with the wave-like rising trend of urban agglomeration development law, which shows a slight wave-like rising trend. Through fitting, the evolutionary rising law of tourism economic resilience in urban agglomerations has been verified, which has universal applicability in urban agglomerations development. Therefore, the evolutionary model of tourism economic resilience can not only be used to analyze and predict the evolutionary trend of tourism economic resilience in urban agglomerations but can also provide planning references and data support for sustainable tourism development in urban agglomerations.

## Discussion

Over the past few years, the topic of narrating and analyzing the sustainable development of tourism has been a hot one in the field of tourism, and the anti-fragility or resilience level of the tourism economy is currently a common entrant. This paper emphasizes the economic nature of the regional tourism industry and argues that tourism resilience should focus on the degree of integration and compatibility between the tourism industry and the regional economy. Therefore, this paper selects the urban agglomeration, the main spatial carrier of regional development, as the research area. Based on the analysis of the dynamic evolution of tourism economic resilience, this paper cross-discussed urban agglomerations development and tourism economic resilience evolution, proposed the principle of the climbing trend of tourism economic resilience in urban agglomerations, confirmed the universal applicability of the climbing trend by simulation and verification. Through this research, it is hoped to enrich the hierarchy of tourism economic resilience research and fill the gaps in the temporal and spatial extension at the current stage.

After considering the spatial–temporal conditions, this paper proposed that the tourism economic resilience follows the spatial economic development law, which moves towards equilibrium in the agglomeration process. Urban agglomerations with relatively strong economic foundations, such as BTH, YRD, and PRD, exhibit higher levels of resilience, creating a significant gap compared to other urban agglomerations. This conclusion complies with the overall polarized trend of provincial tourism economic resilience evaluation in China under the impact of the COVID-19 pandemic, as summarized by Zhang et al.^25^. But within the region, Zhang et al., (2022) believe that the differences in resilience will show an expanding trend, which differs from the conclusion of this article. This may be due to the fact that the concept of resilience emphasized by Zhang et al. (2022) were focused on the defense, recovery and reconstruction of the tourism economy after the epidemic, while the concept of resilience emphasized in this article focuses on the degree of integration and compatibility between the tourism industry and the regional economy. Under the concept of this article, the tourism economic resilience in urban agglomerations shows a gradually balanced trend among regions. The difference in the index selection and the differences in the research areas have led to a divergence in the conclusions between the two articles.

After observing the fitting effect, this paper proposed that the tourism economic resilience conforms to the wave rising trend of the development law in urban agglomeration. From the perspective of urban agglomerations, Mu et al. (2022) cross-discussed urban agglomeration development and the climbing law of urban resilience curve of urban agglomerations, which also concluded that the resilience level is basically manifested as a wave climbing trend^56^. By comparing the simulation and verification results of the two articles, the trend of the development curve is relatively consistent, and the principle of climbing law has substantial similarities. It indicates that the resilience climbing law proposed in this paper conforms to the national characteristics and regional development reality in China, which is suitable for urban agglomerations development. It can provide planning reference and data support for the sustainable tourism development of urban agglomerations.

In addition, this paper constructs a system of tourism economic resilience indicators from four aspects: supply, demand, structure, and management, which are very helpful in assessing the tourism economy resilience to the epidemic. First, the supply resilience aspect. After the outbreak of the epidemic, regions with high tourism supply resilience can quickly adapt to the new market demand to adjust tourism products and provide more health and safety protection. In this conclusion, it can mitigate the impact of supply chain disruptions and maintain the continuity of the tourism business. Second, the demand resilience aspect. Tourism demand may fall during an epidemic, but regions with high demand resilience may be able to attract different types of tourists after the epidemic. In this way, it helps mitigate the impact of demand shocks on tourism. Third, the structural resilience aspect. A diversified tourism structure will likely make urban agglomerations more resilient in their tourism economies, as they will not be exclusively dependent on one type of tourism. Fourth, the managerial resilience dimension. Highly effective tourism management and policy support can ensure that urban agglomerations adapt to future uncertainty and are more likely to energize the tourism industry after an epidemic. Assessing the resilience of tourism economies can help urban agglomerations improve their tourism economies to increase their ability to cope with epidemics or other external shocks and ensure the tourism industry's sustainability and resilience.

Finally, it is important to note that recent research the tourism filed has gradually begun to conduct a quantitative analysis of the epidemic impact on the tourism economic resilience and the reconstruction of the tourism economy^22^,^25^. However, these articles primarily focus on the national or provincial level, with relatively large granularity. Based on data at the city level, this article selects urban agglomerations as the research scope and aims to show the economic attributes of the tourism industry and the compatibility of the tourism economy with the regional economy, thereby reflecting the resilience and anti-fragility of the tourism economy under uncertain risks. However, compared with the national and provincial scales, there is a disadvantage of relatively insufficient data acquisition width and breadth. When major public issues such as epidemics occur, whether the tourism economic resilience development pattern of tourist destinations will deviate from the trend due to mobility restrictions and whether the previously simulated resilience level can withstand the test of special events such as epidemics, this article temporarily lacks data support to provide a more comprehensive empirical explanation. Therefore, regarding the reconstruction of tourism economic resilience development during the epidemic and even in the post-epidemic period, the author of this article hopes that more scholars in the fields of geography, data science, economic statistics, and public health management will join the discussion and conduct interdisciplinary research to provide more reference basis for research on tourism economic resilience in China or even globally.

## Conclusions and policy suggestions

This article uses the method of combined dynamic analysis to measure the tourism economic resilience in urban agglomerations quantitatively. Through spatial kernel density, the spatial effects and patterns of tourism economic resilience in urban agglomerations are observed from a spatial dynamic perspective. Finally, the development curve of the resilience is scientifically verified through a simulation model of evolutionary laws, confirming the universal applicability of resilience evolution trend. Here are the main conclusions:

Firstly, national-level urban agglomerations have a higher level of tourism economic resilience, while non-national-level urban agglomerations have a more robust tourism economic resilience. Upon observation, high resilience level urban agglomerations have gradually achieved stable average annual growth rates due to the more robust economic foundations and larger scales. The urban agglomerations with high growth rates are mostly limited by factors such as location factors, lack of tourism resources, or poor regional spatial development benefits, resulting in poor resilience foundation and still in the early stages of wild growth. In general, the distribution pattern and evolutionary trend of the tourism economic resilience exhibit a substantial similarity to the development laws of the current regional economy in China.

Secondly, the development of tourism economic resilience in urban agglomerations also follows the spatial economic development pattern, which moves towards equilibrium in the aggregation process. Upon observation, the upward trend of regional tourism economic resilience gradually extends from national-level urban agglomerations in the eastern coastal regions to regional and prefecture-level urban agglomerations in the central and western regions. Within the regions, the development of tourism economic resilience gradually radiates from the central cities towards the surrounding cities. In general, the formation and development of urban agglomeration correlate with the rising trend of regional tourism economic resilience.

Thirdly, the tourism economic resilience has a fluctuation climbing node, generally presenting as a wave-like upward trend with fluctuations and stages. Upon observation, national-level urban agglomerations benefit from advantages in geographic location, economic structure, and the depth and breadth of tourism economies, resulting in the highest level of stability in resilient development. However, the regional-level and prefecture-level urban agglomerations are prone to negative spillover effects and easily encounter the development bottlenecks when the resilience level reaches a certain level. This is the change node where the tourism economic resilience of urban agglomeration fluctuates.

Fourthly, the evolutionary trend of tourism economic resilience in urban agglomerations presents a slight wave-like upward curve that changes with time and co-opetition. Upon observation, all the evolutionary curves of the tourism economic resilience have a good fitting effect. In terms of the overall trend, it complies better with the wave-like rising trend of urban agglomeration development law, which shows a slight wave-like rising trend. Through fitting, the evolutionary rising law of tourism economic resilience in urban agglomerations has been verified, which is suitable for developing urban agglomerations.

Considering the above research conclusions and the tourism economy resilience development, the following policy recommendations are offered in order to realize the transformation of the tourism economy’s sustainable development and enhance the comprehensive competitiveness of the tourism industry in Chinese urban agglomerations.

Firstly, multiple parties should work together to enhance the tourism economy resilience of urban agglomerations. To begin with, it should improve the tourism economy resilience of urban agglomerations from the supply side. For example, strengthen the construction of hotels and tourist attractions, especially star-rated hotels and scenic spots. In this way, it can create more jobs in the tourism economy industry and improve the quality of public services. Next, it should understand and satisfy consumer expectations and energize tourism consumption from the demand side. Then, continuously optimize the structure of the tourism industry and form the industrial layout with complementary advantages between cities. Strengthen the construction of tourism digitization, such as applying virtual digital person technology to digital tour guides. Finally, strive to form the organic unity of the market and t the government, which can promote the high-quality development of tourism economic resilience. The two are complementary and work together to encourage each other.

Secondly, cross-city cooperation and linkages can jointly improve the overall tourism economic resilience to reduce social risks’ impact. When a liquidity crisis or epidemic occurs, the spatial shift between long-distance and short-distance tourists can provide a resilient living space for the tourism economy. In the post-epidemic era, the forming of cross-city cooperation and linkage mechanisms is the key to the spatial shift of long-distance and short-distance tourists. On the one hand, establish a tourism cooperation mechanism for urban agglomerations. It creates cross-city tourism cooperation platforms and establishes joint websites or digital platforms. As a result of these approaches, cities are able to exchange information and resources so that they can respond to emergencies quickly and collaboratively. On the other hand, promote online tourism to digitize tourism. During liquidity crises or epidemics, provide tourism content online to maintain interaction with tourists and mitigate the extent of physical tourism.

Thirdly, establish a crisis management mechanism and formulate a comprehensive crisis management plan to enhance the risk-resistant capacity of the tourism economy of urban agglomerations. This mainly responses to public health emergencies and natural disasters. In the first instance, formulate a joint crisis response plan. Urban agglomerations should develop joint crisis response plans in advance, clarify each city’s responsibilities and concerted actions, as well as ensure an orderly response in times of crisis. Alternatively, promote joint insurance programs and establish emergency assistance funds. Tourism enterprises within an urban agglomeration can work together to promote a coordinated insurance program to share the losses caused by the crisis and improve the overall tourism economic resilience. Create an emergency assistance fund for urban agglomerations in the event of a problem to ensure tourists’ immediate financial assistance, and tourism enterprises in times of crisis.

## Data Availability

All the data and materials supporting the results and analyses presented in this paper are available upon request. The datasets generated during and/or analyzed during the current study are available from the corresponding author upon reasonable request.

## References

[1] Weaver D, Tang C, Zhao Y (2020). Facilitating sustainable tourism by endogenization: China as exemplar. Ann. Tour. Res..

[2] Zhang H, Jiang Z, Gao W, Yang C (2022). Time-varying impact of economic policy uncertainty and geopolitical risk on tourist arrivals: Evidence from a developing country. Tour. Manag. Perspect..

[3] Peng YT, Saboori B, Ranjbar O, Can M (2023). Global perspective on tourism-economic growth nexus: The role of tourism market diversification. Tour. Plan. Dev..

[4] Bangwayo-Skeete PF, Skeete RW (2021). Modelling tourism resilience in small island states: A tale of two countries. Tour. Geogr..

[5] Pan SY, Gao M, Kim H, Shah KJ, Pei SL, Chiang PC (2018). Advances and challenges in sustainable tourism toward a green economy. Sci. Total Environ..

[6] Jiao X, Li G, Chen JL (2020). Forecasting international tourism demand: A local spatiotemporal model. Ann. Tour. Res..

[7] Tsui KWH (2017). Does a low-cost carrier lead the domestic tourism demand and growth of New Zealand?. Tour. Manag..

[8] Zeng P, Wei X, Duan Z (2022). Coupling and coordination analysis in urban agglomerations of China: Urbanization and ecological security perspectives. J. Clean. Prod..

[9] Bianco D, Bueno A, Godinho Filho M, Latan H, Ganga GMD, Frank AG, Jabbour CJC (2023). The role of Industry 4.0 in developing resilience for manufacturing companies during COVID-19. Int. J. Prod. Econo..

[10] Reggiani A, De Graaff T, Nijkamp P (2002). Resilience: An evolutionary approach to spatial economic systems. Netw. Spat. Econ..

[11] Welsh M (2014). Resilience and responsibility: Governing uncertainty in a complex world. Geogr. J..

[12] Sellberg MM, Ryan P, Borgström ST, Norström AV, Peterson GD (2018). From resilience thinking to resilience planning: Lessons from practice. J. Environ. Manag..

[13] Filimonau V, De Coteau D (2020). Tourism resilience in the context of integrated destination and disaster management (DM2). Int. J. Tour. Res..

[14] Prayag G (2023). Tourism resilience in the ‘new normal’: Beyond jingle and jangle fallacies?. J. Hosp. Tour. Manag..

[15] Duro J, Perez-Laborda A, Fernandez M (2022). Territorial tourism resilience in the COVID-19 summer. Ann. Tour. Res. Empir. Insights.

[16] Kim S, Bramwell B (2019). Boundaries and boundary crossing in tourism: A study of policy work for tourism and urban regeneration. Tour. Manag..

[17] Kutzner D (2019). Environmental change, resilience, and adaptation in nature-based tourism: Conceptualizing the social-ecological resilience of birdwatching tour operations. J. Sustain. Tour..

[18] Dimelli DP (2017). The effects of tourism in Greek insular settlements and the role of spatial planning. J. Knowl. Econ..

[19] Marco-Lajara B, Úbeda-García M, Ruiz-Fernández L, Poveda-Pareja E, Sánchez-García E (2022). Rural hotel resilience during COVID-19: The crucial role of CSR. Curr. Issues Tour..

[20] Wieczorek-Kosmala M (2022). A study of the tourism industry's cash-driven resilience capabilities for responding to the COVID-19 shock. Tour. Manag..

[21] Tasnim Z, Shareef MA, Dwivedi YK, Kumar U, Kumar V, Malik FT, Raman R (2022). Tourism sustainability during COVID-19: Developing value chain resilience. Oper. Manag. Res..

[22] Ntounis N, Parker C, Skinner H, Steadman C, Warnaby G (2022). Tourism and hospitality industry resilience during the Covid-19 pandemic: Evidence from England. Curr. Issues Tour..

[23] Quang TD, Tran TC, Tran VH, Nguyen TT, Nguyen TT (2022). Is Vietnam ready to welcome tourists back? Assessing COVID-19’s economic impact and the Vietnamese tourism industry’s response to the pandemic. Curr. Issues Tour..

[24] Shi, W., Gong, Y., Wang, L., & Nikolova, N. Heterogeneity of inbound tourism driven by exchange rate fluctuations: Implications for tourism business recovery and resilience in Australia. Curr. Issues Tour. 1–18 (2022).

[25] Zhang P, Huang Y, Pan S, Chen W, Zhong H, Xu N, Zhong M (2022). Does resilience exist in China’s tourism economy? From the perspectives of resistance and recoverability. Sustainability.

[26] Boto-García D, Mayor M (2022). Domestic tourism and the resilience of hotel demand. Ann. Tour. Res..

[27] Tseng YP, Huang YC, Li MS, Jiang YZ (2022). Selecting key resilience indicators for Indigenous community using Fuzzy Delphi method. Sustainability.

[28] Della Corte, V., Doria, C., & Oddo, G. The impact of COVID‐19 on international tourism flows to Italy: Evidence from mobile phone data. The World Economy (2021). 10.1111/twec.13380 PMC9880759 36721456

[29] Lai YL, Cai W (2022). Enhancing post-COVID-19 work resilience in hospitality: A micro-level crisis management framework. Tour. Hosp. Res..

[30] Gottschalk M, Kuntz JC, Prayag G (2022). TouRes: Scale development and validation of a tourist resilience scale. Tour. Manag. Perspect..

[31] Prayag G (2020). Time for reset? COVID-19 and tourism resilience. Tour. Rev. Int..

[32] Mori T, Smith TE (2015). On the spatial scale of industrial agglomerations. J. Urban Econ..

[33] Fang C, Yu D (2017). Urban agglomeration: An evolving concept of an emerging phenomenon. Landsc. Urban Plan..

[34] Fang C, Liang L, Wang Z (2019). Quantitative simulation and verification of upgrade law of sustainable development in Beijing-Tianjin-Hebei urban agglomeration. Sci. China Earth Sci..

[35] Gan C, Voda M, Wang K, Chen L, Ye J (2021). Spatial network structure of the tourism economy in urban agglomeration: A social network analysis. J. Hosp. Tour. Manag..

[36] Liu Z, Geng Y, Ulgiati S, Park HS, Tsuyoshi F, Wang H (2016). Uncovering key factors influencing one industrial park's sustainability: A combined evaluation method of emergy analysis and index decomposition analysis. J. Clean. Prod..

[37] Yan Y, Zhou R, Ye X, Zhang H, Wang X (2018). Suitability evaluation of urban construction land based on an approach of vertical-horizontal processes. ISPRS Int. J. Geo-Inform..

[38] Miao W, Pan S, Sun D (2016). A rank-corrected procedure for matrix completion with fixed basis coefficients. Math. Program..

[39] Quah D, T.  (1997). Empirics for growth and distribution: stratification, polarization, and convergence clubs. J. Econ. Growth.

[40] Duan Z, Tang T (2022). Quantitative simulation and verification of the coordination curves between sustainable development and green innovation efficiency: From the perspective of urban agglomerations development. Sustainability.

[41] Fang C, Liang L, Chen D (2022). Quantitative simulation and verification of urbanization and eco-environment coupling coil in Beijing-Tianjin-Hebei urban agglomeration, China. Sustain. Cities Soc..

[42] Niu F, Yang X, Wang F (2020). Urban agglomeration formation and its spatiotemporal expansion process in China: From the perspective of industrial evolution. Chin. Geogr. Sci..

[43] Mtapuri O, Camilleri MA, Dłużewska A (2022). Advancing community-based tourism approaches for the sustainable development of destinations. Sustain. Dev..

[44] Czarnecki A, Dacko A, Dacko M (2023). Changes in mobility patterns and the switching roles of second homes as a result of the first wave of COVID-19. J. Sustain. Tour..

[45] Arbulú I, Razumova M, Rey-Maquieira J, Sastre F (2021). Measuring risks and vulnerability of tourism to the COVID-19 crisis in the context of extreme uncertainty: The case of the Balearic Islands. Tour. Manag. Perspect..

[46] Cró S, Martins AM (2017). Structural breaks in international tourism demand: Are they caused by crises or disasters?. Tour. Manag..

[47] Calgaro E, Lloyd K, Dominey-Howes D (2014). From vulnerability to transformation: A framework for assessing the vulnerability and resilience of tourism destinations. J. Sustain. Tour..

[48] Prayag G (2018). Symbiotic relationship or not? Understanding resilience and crisis management in tourism. Tour. Manag. Perspect..

[49] Sharma GD, Thomas A, Paul J (2021). Reviving tourism industry post-COVID-19: A resilience-based framework. Tour. Manag. Perspect..

[50] Lu R, Yang Z (2023). Analysis on the structure and economic resilience capacity of China’s regional economic network. Appl. Econ..

[51] Anguera-Torrell O, Cerdan A (2021). Which commercial sectors coagglomerate with the accommodation industry? Evidence from Barcelona. Cities.

[52] Ketter E (2022). Bouncing back or bouncing forward? Tourism destinations’ crisis resilience and crisis management tactics. Eur. J. Tour. Res..

[53] Wang J, Huang X, Gong Z, Cao K (2020). Dynamic assessment of tourism carrying capacity and its impacts on tourism economic growth in urban tourism destinations in China. J. Destin. Mark. Manag..

[54] Chen J, Gao M, Cheng S, Hou W, Song M, Liu X, Liu Y (2022). Global 1 km × 1 km gridded revised real gross domestic product and electricity consumption during 1992–2019 based on calibrated nighttime light data. Sci. Data.

[55] Xiao Y, Tang X, Wang J, Huang H, Liu L (2022). Assessment of coordinated development between tourism development and resource environment carrying capacity: A case study of Yangtze River economic Belt in China. Belt in China. Ecol. Indic..

[56] Mu X, Fang C, Yang Z (2022). Spatio-temporal evolution and dynamic simulation of the urban resilience of Beijing-Tianjin-Hebei urban agglomeration. J. Geogr. Sci..

